# Supplementary material for: Predicting visual function by interpreting a neuronal wiring diagram
Source: Nature. 2024 Oct 2;634(8032):113–23. doi: 10.1038/s41586-024-07953-5 (PMC11446822; doi:10.1038/s41586-024-07953-5)
Supplement: Supplementary file 6 — CRF and ERF predictions for individual TmY4 and TmY9 cells. Analogous to Supplementary Data 3, but for TmY target types. Shown are the top four monosynaptic pathways, the strongest pathway passing through each of the top ten intermediary types (ranking from Extended Data Fig. 7), and the trisynaptic pathway Tm1–TmY–Dm3–TmY (see the section entitled Prediction of spatial normalization). [file 41586_2024_7953_MOESM6_ESM.zip › DataS4/TmY9qΓèÑ/720575940621595312.pdf]

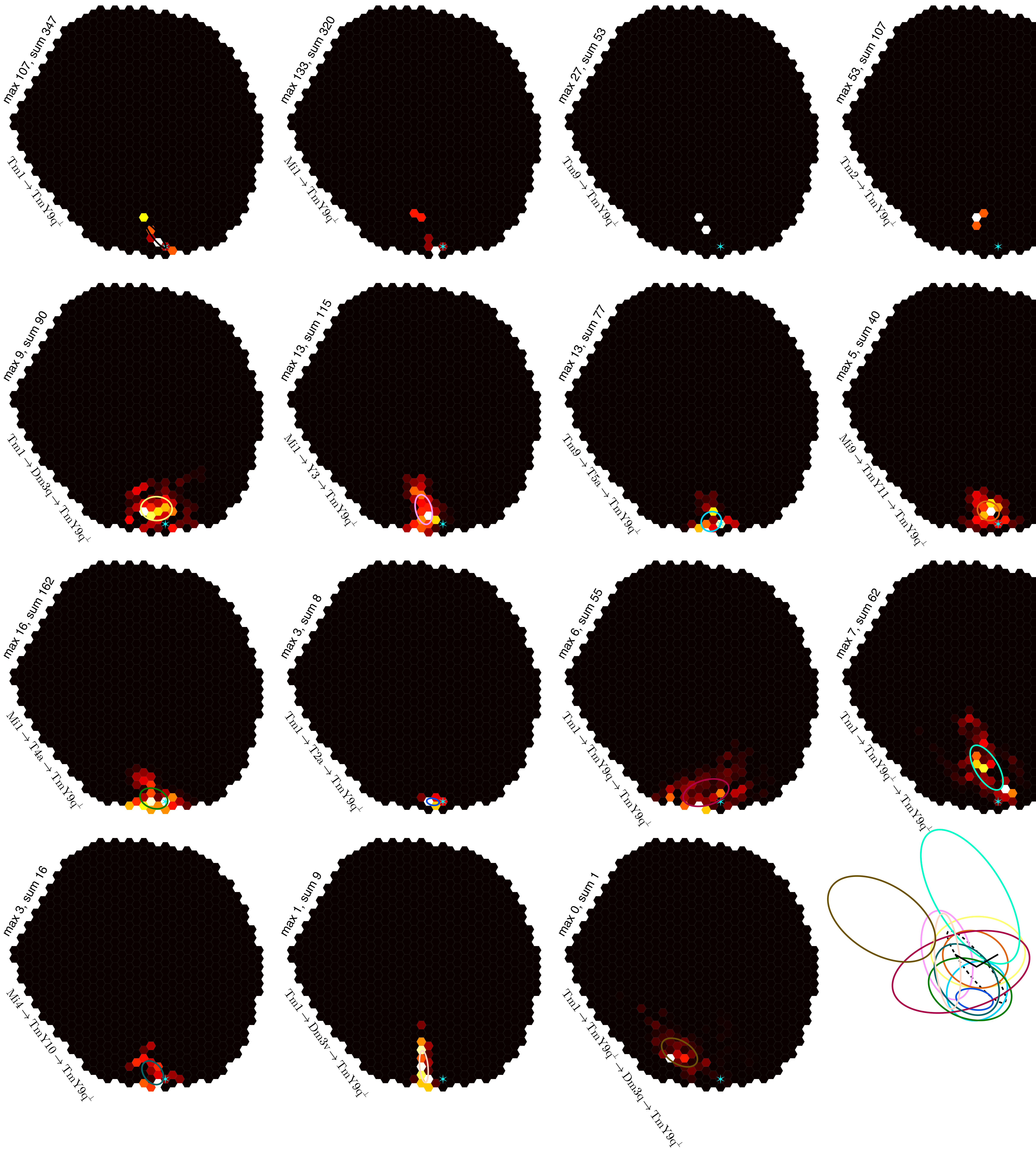

max 107, sum 347
Tm1 → TmY9q⊥
max 133, sum 320
Mi1 → TmY9q⊥
max 27, sum 53
Tm9 → TmY9q⊥
max 53, sum 107
Tm2 → TmY9q⊥
max 9, sum 90
Tm1 → Dm3q → TmY9q⊥
max 13, sum 115
Mi1 → Y3 → TmY9q⊥
max 13, sum 77
Tm9 → T5a → TmY9q⊥
max 5, sum 40
Mi9 → TmY11 → TmY9q⊥
max 16, sum 162
Mi1 → T4a → TmY9q⊥
max 3, sum 8
Tm1 → T2a → TmY9q⊥
max 6, sum 55
Tm1 → TmY9q → TmY9q⊥
max 7, sum 62
Tm1 → TmY9q⊥ → TmY9q⊥
max 3, sum 16
Mi4 → TmY10 → TmY9q⊥
max 1, sum 9
Tm1 → Dm3v → TmY9q⊥
max 0, sum 1
Tm1 → TmY9q⊥ → Dm3q → TmY9q⊥
